# Cranial bone morphometric study among mouse strains

**DOI:** 10.1186/1471-2148-8-73

**Published:** 2008-02-29

**Authors:** Minoru Kawakami, Ken-ichi Yamamura

**Affiliations:** 1 Division of Developmental Genetics, Institute of Molecular Embryology and Genetics, Kumamoto University, 2-2-1, Honjo, Kumamoto City, Kumamoto, 860-0811, Japan

## Abstract

**Background:**

Little is known about the molecular mechanism which regulates how the whole cranium is shaped. Mouse models currently available for genetic research include several hundreds of unique inbred strains and genetically engineered mutants.

By cross comparing their genomic structures, we can elucidate the cause of any differences in the phenotype between two strains. The craniometry of subspecies, or closely related species, of mice provide a good systemic model to study the relationship between genetic variance and cranial shape evolution. The lack of a quantified framework for comparing and analyzing mouse cranial shape has been a problem. For this reason, we performed quantitative analysis of cranial shape morphology between several mouse strains.

**Results:**

This article reports on a craniometric assay of seven mouse strains: four inbred strains (C57BL/6J, BALB/cA, C3H/HeJ, and CBA/JNCr) from *Mus musculus domesticus *(*M. m. domesticus*); one closed colony strain (ICR) from *M. m. domesticus*; one inbred strain (MSM/Ms) from *Mus musculus molossinus*; and, *Mus spretus *as a strain from a species other than *M. m. domesticus*. We performed linear measurements and geometric morphometrics. Geometric morphometrics revealed that the cranial characteristics of each strains were clearly distinguishable. We obtained mean scores for each species using the tpsRelw Program and plotted them.

**Conclusion:**

Geometric morphometrics proved to be useful for identifying and classifying variations in form, and it revealed that *M. spretus *has a slender cranium when compared with our other strains. The mean cranial shape of C3H or CBA was more similar to MSM/Ms, which is derived from *M. m. molossinus*, than to either C57BL/6J, BALB, or ICR which are derived from *M. m. domesticus*. Future work in this field will aid in elucidating the mechanism of whole cranial shape regulation.

## Background

Over 450 inbred mouse strains have been described and developed up until now, providing plentiful phenotypes and genomic backgrounds for genetic studies. Most inbred laboratory strains are known to have originated from a limited founder population of *Mus musculus musculus *and *M. m. domesticus *housed within a small number of research facilities and laboratories [1]. Most of these strains have been bred for over 150 generations; they are isogenic and homogeneous for over 98.6% of their genomes [2]. In the past few years, there have been several articles on the phylogenetic relationships among these inbred strains based on the available genome data. Wade et al reported on the fine structure of variation in the mouse genomes using SNPs [3]. They suggested these genomes to be mosaics, most being derived from *M. m. domesticus *and a minority from other subspecies. Recently, Sakai et al used the information from more than 1200 microsatellite loci to refine the fine phylogenetic relationships among inbred strains [1].

Here, we studied craniometric relationships among mouse strains. The mouse cranium has many significant features. Firstly, like all vertebrate crania, it is a complex structure comprising and protecting many important organs: brain; jaws; and, sensory organs, such as the eyes, the ears, and the nose. It is therefore appropriate and logical that the morphological diversity of the cranium should reflect phylogenetic and functional aspects of the adaptive evolution of each species after speciation. Secondly, the morphology of hard tissues, such as cranial bones, is easily measured and compared. Thirdly, the cranium is constructed from multiple bony components: the developmental process of the formation of the bones, and the sutures between them, has already been described in detail [4-6]. Fourthly, it is already known that mutations at many loci affect craniometric characteristics [7]. Considering the above, it would seem reasonable to assume that the craniometry of subspecies, or of closely related species, of mice provides a good systemic model for studying the relationship between cranial evolution and genetic variance.

The main purpose of this paper is to find whether the craniometric relationship and the phylogenic pattern, which is obtained from genomic data, among our subject species, correspond or not. To solve this question, the first thing that was necessary to be done was to select an appropriate method to quantify the cranial morphology of each sample. Next, we had to examine whether utilizing this method we could identify the quantitative differences among those species.

Questions concerning the extent and nature of morphological diversity, among closely related species and subspecies, are best addressed within a morphospace [8], and geometric morphometrics is a powerful tool for providing a morphospace for studying this diversity. Traditional multivariate methods are known to have weaknesses [9,10]. Relative warp analysis, which is a technique within the family of geometric morphometrics, offers a useful way to quantify shape variation independently of variation in the three parameters: size; translation; and, orientation. Algorithms for calculating relative warps are given by Bookstein [10] and Rohlf [11]. In this approach, landmark coordinates for all the specimens are superimposed using Procrustes methods [12], such that the fit between all the specimens is as close as possible. A reference specimen is computed and established as the mean of the fitted landmark coordinates. Relative warp analysis can be performed using the tpsRelw program by F. J. Rohlf [13]. We used geometric morphometrics in a craniometric assay of several mouse strains, subspecies and species: it was revealed that the cranial characteristics of each strain, subspecies or species were clearly distinguishable.

## Results

### Linear Distance Measurements

**Figure 1:**
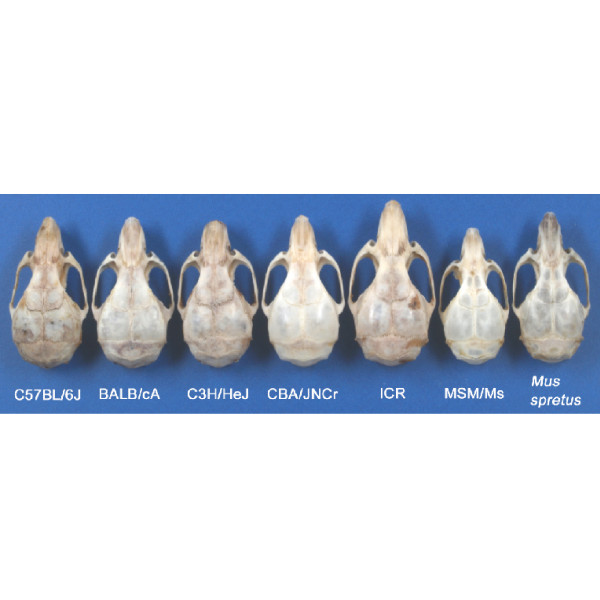
Cranial Shape Variations in Seven Mouse Strains (Female, 12 Weeks Old).

**Figure 2:**
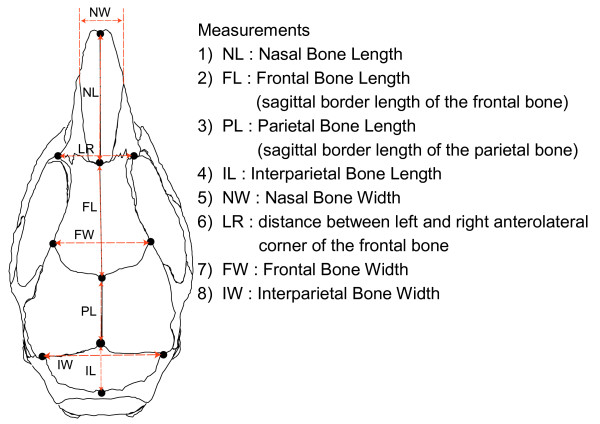
Linear Measurements of the Cranium.

Figure 1 shows an example of a cranium from each strain (female, 12 weeks old). The crania of the different strains differ from each other in their general appearance. We first performed a preliminary morphometric study using conventional linear distance measurements. We placed 11 landmarks on the cranium, based upon which we made eight measurements (Fig. 2). These landmarks were located on the boundaries between the bones, of different parts of the cranium, for measuring the dimensions of these bones. Table 1 shows the results of linear distance measurements for each sample. The standard deviations of the measurements indicate that intraspecies variations in the specimens are sufficiently small.

**Table 1:** Summary of the Linear Measurements Among Mouse Strains.

No.	Strain	Sex	distance (mm)							
			
			NL	FL	PL	IL	NW	LR	FW	IW
1	C57BL/6J	♂	7.7	7.7	4	3.6	2.7	5	5.3	8.4
2			7.6	7.6	4	3.5	2.6	5.1	5.5	8.3
3			7.4	7.8	4	3.5	2.6	5	5.3	8.1
4			7.8	7.8	4.1	3.5	2.7	5.2	5.4	8.1
5			7.8	7.8	3.7	3.5	2.6	5.1	5.4	8
6			7.6	7.8	4	3.7	2.7	5.1	5.3	8.4
7		♀	7.6	7.8	3.8	3.7	2.6	5.1	5.3	7.8
8			7.1	7.8	3.7	3.6	2.5	5.2	5.5	7.8
9			7.7	7.7	3.7	3.5	2.6	5.1	5.2	8

Mean			7.589	7.756	3.889	3.6	2.622	5.1	5.356	8.1
SD			0.22	0.073	0.162	0.1	0.067	0.071	0.101	0.229

10	BALB/cA	♂	8	7.2	4.8	3.1	2.6	5	5.1	9
11			8.3	7.1	5.1	3.1	2.5	5.1	5	9
12			8.1	7.3	5.1	2.9	2.7	5	5.2	9
13		♀	7.1	6.3	4.7	2.9	2.5	4.8	5.5	8.6
14			7.8	6.8	4.6	3.0	2.5	5	5.1	8.9
15			8.2	6.6	4.6	3.0	2.5	4.8	5.1	8.7

Mean			7.917	6.883	4.817	3.0	2.55	4.95	5.167	8.867
SD			0.436	0.387	0.232	0.1	0.084	0.122	0.175	0.175

16	C3H/HeJ	♂	7.5	7	4.9	3.3	2.5	5	4.6	8.8
17			7.3	7	4.8	3.4	2.5	5	4.8	8.8
18			7.3	7.1	4.8	3.4	2.6	4.9	4.7	8.8
19		♀	7.5	7.1	4.8	3.4	2.5	4.9	5	8.9
20			7.2	6.6	4.6	3.4	2.7	4.9	5.1	8.7
21			7.7	7	4.6	3.4	2.6	4.9	5	8.7

Mean			7.417	6.967	4.75	3.3	2.567	4.933	4.867	8.783
SD			0.183	0.186	0.122	0.0	0.082	0.052	0.197	0.075

22	CBA/JNCrj	♂	7.2	7.2	4.6	3.7	2.3	5.1	5.2	8.6
23			7.5	7.7	4.5	3.7	2.3	5.1	5.1	8.6
24			7.5	7.4	4.5	3.7	2.3	5.1	5	8.6
25		♀	7.6	7.5	4.3	3.9	2.4	5.1	5.3	8.7
26			7.6	7.5	4.3	3.8	2.4	5	5.1	8.6
27			7.7	7.5	4.3	3.8	2.4	5	5.1	8.8

Mean			7.517	7.467	4.417	3.8	2.35	5.067	5.133	8.65
SD			0.172	0.163	0.133	0.1	0.055	0.052	0.103	0.084

28	ICR	♂	8	7.4	4.4	3.2	2.9	5.2	5.1	8.6
29			7.8	7.4	4.4	3.2	2.8	5.4	5	8.6
30			8.1	7.4	4.4	3.2	2.7	5.4	5	8.6
31		♀	8.8	7.4	4.4	3.4	2.9	5.4	5	8.6
32			8.8	7.2	4.5	3.5	2.7	5.4	4.8	8.5
33			8.4	7.4	4.5	3.2	2.9	5.5	4.8	8.5

Mean			8.317	7.367	4.433	3.3	2.817	5.383	4.95	8.567
SD			0.422	0.082	0.052	0.1	0.098	0.098	0.122	0.052

34	MSM/Ms	♂	6.8	5.4	4.4	3.0	2.1	4.4	4.1	7.2
35			6.9	5.4	4.4	3.1	2.2	4.3	4.1	7.2
36			6.7	5.8	4.6	3.0	2.2	4.3	4.1	7.2
37			6.7	5.8	4.5	3.1	2.1	4.3	4.1	7.2
38		♀	6.7	5.2	4.5	3.1	2.1	4.3	4	7.2
39			6.7	5.7	4.4	3.2	2.1	4.4	3.9	7.2
40			6.8	5.7	4.3	3.2	2.2	4.4	3.8	7.2
41			6.8	5.8	4.6	3.1	2.2	4.5	3.9	7.2

Mean			6.763	5.6	4.463	3.1	2.15	4.363	4	7.2
SD			0.074	0.233	0.106	0.08	0.053	0.074	0.12	9E-16

42	*M. spretus*	♂	8	6	4.7	3.4	2.3	4.2	4.7	7.2
43			8	6.1	4.9	3.3	2.1	4.6	4.3	7.2
44			7.9	6.1	4.7	3.3	2.2	4.6	4.6	7.2
45			8.4	6	4.6	3.4	2.2	4.7	4.4	7.2
46		♀	8	6.1	4.8	3.4	2	5	4.3	7.2
47			7.8	5.8	4.8	3.3	2.4	4.8	4.2	7.2
48			8.2	5.9	4.8	3.3	2.1	5	4.3	7.2
49			8.3	5.9	4.8	3.3	2.1	4.8	4.4	7.3

Mean			8.075	5.988	4.763	3.3	2.175	4.713	4.4	7.213
SD			0.205	0.113	0.092	0.0	0.128	0.259	0.169	0.035

NL, Nasal Bone Length; FL, Frontal Bone Length (sagittal border length of frontal bone); PL, Parietal Bone Length (sagittal border length of parietal bone); IL, Interparietal Bone Length; NW, Nasal Bone Width; LR, distance between left and right anterolateral corner of the frontal bone; FW, Frontal Bone Width; IW, Interparietal Bone Width.

### Morphospace Axes

**Figure 3:**
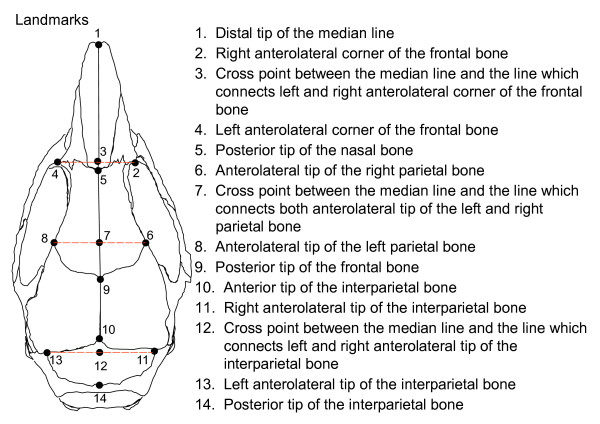
Landmarks Used to Describe the Cranial Shape in Geometric Morphometric Analyses.

It is not easy to integrate the results of linear measurements analyses into a comparative morphological study systematically. We therefore performed morphological comparisons, with shape variables, using geometric morphometric methods [10]. We photographed all specimens in dorsal view, using a Keyence Digital Microscope; thusly, 14 biologically homologous cranial landmarks were digitized (Fig. 3).

**Figure 4:**
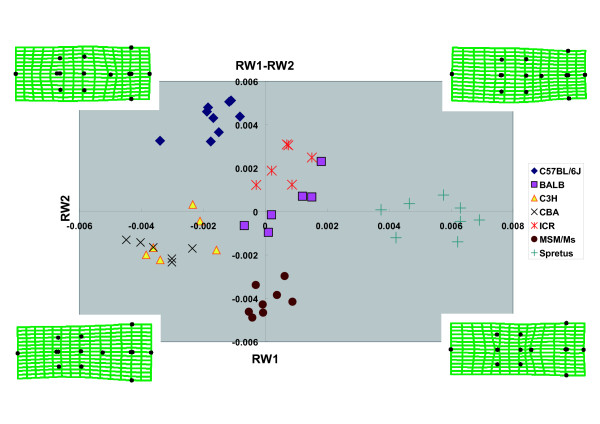
**Scatter Plot of Scores on Relative Warps 1 and 2**. Deformation grids indicate general shapes of each quadrant.

**Figure 5:**
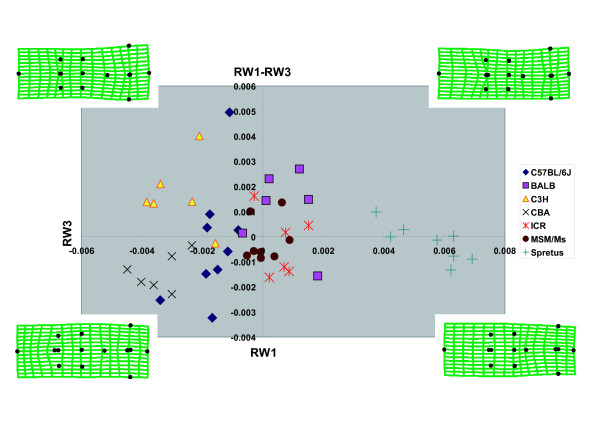
**Scatter Plot of Scores on Relative Warps 1 and 3**. Deformation grids indicate general shapes of each quadrant.

**Figure 6:**
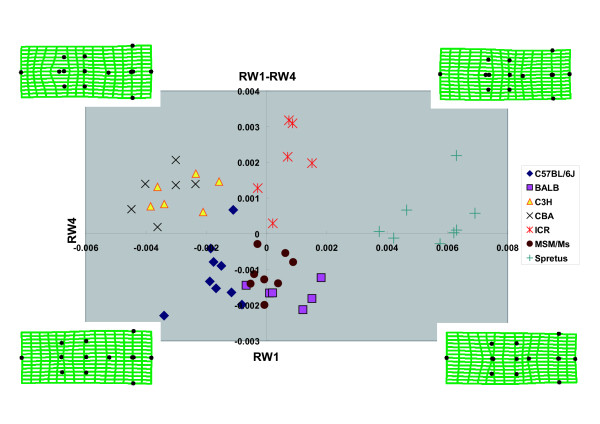
**Scatter Plot of Scores on Relative Warps 1 and 4**. Deformation grids indicate general shapes of each quadrant.

Four relative warp axes (RW1-4) together accounted for 75.1% of the total shape variation in the data. The major axes of shape variation in the crania of the seven strains can be seen in the plot (Fig. 4) of all specimens on RW1 and RW2 which accounted for 30.6% and 28.0%, respectively, of all variation in the samples. Significant interspecific clustering was observed in this morphospace. RW1 accounted for 30.6% of the variance and described the length of the frontal bone and the sphenoidal angle of the parietal bone (Fig. 4). *M. spretus *was characterized by relatively short frontal bones and a short sphenoidal angle of the parietal bone; in contrast, CBA possessed relatively long frontal bones and a long sphenoidal angle of the parietal bone. RW2 accounted for 28.0% of the variance and described the width of the frontal bone (Fig. 4). C57BL/6J had relatively wide frontal bones in contrast to the narrow frontal bones of MSM. RW3 accounted for 9.1% of the variance and described the length of the nasal bone (Fig. 5). BALB/cA was characterized by relatively long nasal bones when compared with CBA/JNCr. The axes RW1 and RW3 together discriminated C3H (positive value) and CBA (negative value). RW4 accounted for 7.4% of the variance and described the width of the region around the nasal border of the frontal bone (Fig. 6). This width was wider in ICR, and narrower in MSM. The axes RW1 and RW4 together discriminated ICR (large positive value) and BALB (large negative value).

### Correlation Between Relative Warp and Centroid Size

Shape and size are correlated in many cases. So we performed analysis to find the precise correlation between size and shape. Analyses of cranial size used the standard measure of geometric scale, which is centroid size (CS), defined as the square root of the squared distance between each landmark and the centroid of the landmark configurations summed over all landmarks. Then we performed a univariate regression analysis between each of the first 4 relative warps and CS (Table 2).

**Table 2:** Summary of the Regression Analysis of the First 4 RW Scores on the Centroid Size.

	R	*F*	Probability
RW1	0.315374	5.190951	0.027293
RW2	0.576152	23.35412	1.48E-05
RW3	0.017629	0.014612	0.904302
RW4	0.388199	8.339589	0.005847

RW, relative warp; R, correlation coefficient

**Figure 7:**
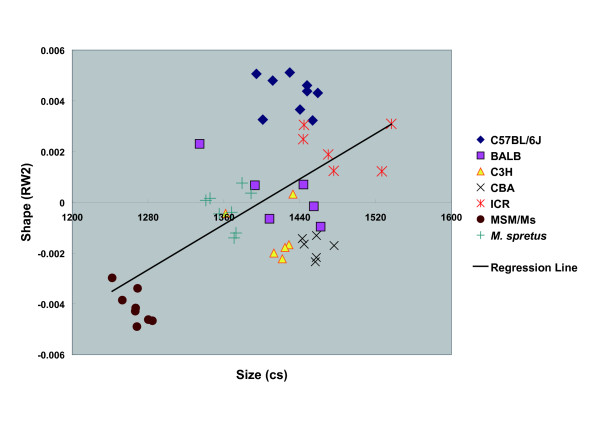
**Regression of Relative Warp 2 Scores on Centroid Size**. Scatterplot summarizing relations between the size (centroid size [CS]) and shape (relative warp 2 [RW]) variation. Regression (n = 49) of RW2 scores on CS (*F *= 23.35412; *P *= 1.48E-05; R^2 ^= 0.331951).

**Figure 8:**
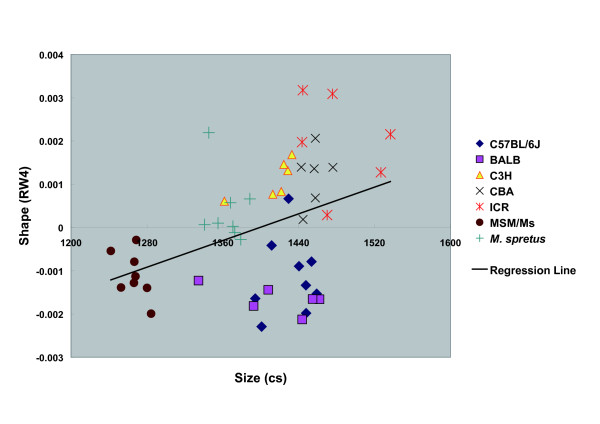
**Regression of Relative Warp 4 Scores on Centroid Size**. Scatterplot summarizing relations between the size (centroid size [CS]) and shape (relative warp 4 [RW]) variation. Regression (n = 49) of RW4 scores on CS (*F *= 8.339589; *P *= 0.005847; R^2 ^= 0.150698).

RW2 produced a significant regression on CS (*P *= 1.48E-05, R = 0.576152) (Fig. 7), indicating that the RW2 axis largely describes shape variation associated with size. RW4 produced a weak regression on CS (*P *= 0.005847, R = 0.388199) (Fig. 8). Regressions of RWs 1 or 3 on CS were not significant (*P *> 0.05). In terms of size, MSM/Ms animals are smaller than are the others, and they have relatively small RW2 values (Fig. 7).

### Cluster Analyses

**Figure 9:**
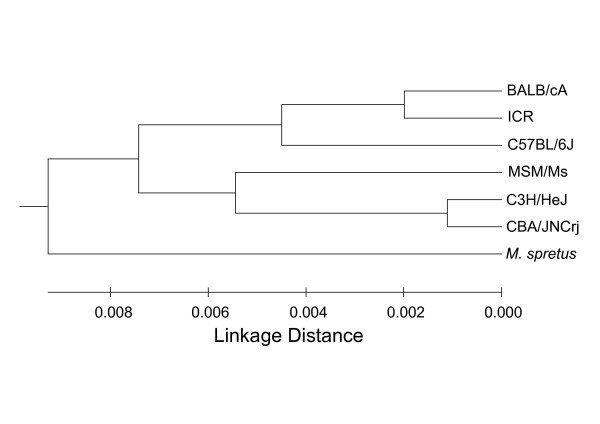
**Cluster Analysis of the Mean Cranial Shape of Each Species**. Cluster analysis based on the distance between mean cranial shape of each species on the scatter plot for relative warps 1 and 2.

We obtained mean scores for each species using the tpsRelw Program and plotted them in RW1 and RW2. Based on the mean distance between species, we generated a phylogenic tree using the UPGMA Method (Fig. 9). This tree reflects morphological similarity between species; however, only a part of this pedigree matches the tree that was created from the genomic sequence (for example C3H and CBA), whereas some parts of this pedigree did not match the genomic sequence tree.

## Discussion

The current classical laboratory strains were derived (in unequal percentages) from three parental subspecies: *Mus musculus domesticus*; *M. m. musculus*; and, *M. m. castaneus*. *M. m. domesticus *is common in Western Europe, Africa, and the Near East [14]. The range of *M. m. musculus *spans from Eastern Europe to Japan, across Russia and northern China. *M. m. castaneus *is found from Sri Lanka to South-East Asia, including the Indo-Malayan archipelago. None of these subspecies was completely isolated genetically, and in the common regions there is evidence of genetic exchange ranging from limited introgression to complete blending. Such exchanges have occurred between *M. m. musculus *and *M. m. domesticus *in Europe, and between *M. m. musculus *and *M. m. castaneus *in China and Japan. In Japan, *M. m. musculus *and *M. m. castaneus *have hybridized extensively and have given rise to a unique population referred to as *Mus musculus molossinus *[15,16]. *M. spretus *is the well-known western Mediterranean short-tailed mouse, a different species of the genus *Mus*.

In this study, we performed geometric morphometrics, a method developed by Fink and Zelditch (1995)[17]. There are three main points with respect to these results to take note of. Firstly, the cranial morphology of each strain is clearly distinguishable by geometric morphometrics. RW1 and RW2 discriminated among C57BL/6J, MSM/Ms, and *M. spretus*, although C3H overlapped with CBA and BALB overlapped with ICR. RW1 and RW3 discriminated between C3H and CBA, and RW1 and RW4 distinguished ICR from BALB. Secondly, according to our morphometric data, the mean cranial shapes of C3H and CBA were morphologically close to each other, at least with respect to RW1 and RW2 which represent the major axes of shape variation in the crania of the seven strains we studied. Strains C3H and CBA are considered genealogically closely related [18] and a small genetic distance between them has been demonstrated by SNP analysis [19]. On the other hand, *M. spretus*, a different species from the other six strains, showed a quite different mean cranial shape. Thirdly, our data also suggests that genetically close strains do not always possess morphologically similar crania; for example, the mean cranial shape of C3H and CBA were more similar to MSM/Ms, which is derived from *M. m. molossinus *[20], than to either C57BL/6J, BALB, or ICR which are paradoxically genetically closer strains. There are several possible explanations for this inconsistency. The first is that the genomic regions, which specifically control cranial morphology, are well conserved compared with the overall low homology of genomes between two genetically distant but morphologically similar species: in this case, only small regions are synapomorphic. The second possibility is that some epigenetic factors affect the morphology. The third possibility is that the resemblance of cranial morphology is caused by homoplasy: in this case, functional factors predominate over phylogenetic factors. The fourth, unknown environmental effects can also account for the results.

There have been craniometric studies in mice or other animals by several groups. Hallgrímsson et al (2004) reported that A/WySnJ mice had different craniofacial shapes compared with C57BL/6J mice [21]. Klingenberg et al (2004) compared the mandibles between the strains LG/J and SM/J utilizing geometric morphometrics [22]. In this study, we analyzed seven strains, and our results are applicable to comparative morphological studies of mice. Mutations in *cis*-regulatory sequences have been implicated as being the predominant source of variation in morphological evolution. Fondon and Garner (2004) reported that variation in repeats, in coding regions, is associated with morphology [23]. In the case of cichlid fish, some loci are significantly associated with jaw shape [24]. It is possible that some of our results from geometric morphometrics could be explained by association with some particular genomic structure. It is also possible that the epigenetic factors or functional factors could account for the results. For example, epigenetic interactions were reported by Hallgrímsson et al (2007)[25], and functional effects were analyzed by Zelditch et al (2004, 2006)[26,27], or Willmore et al (2006)[28]. Recently, there have been many reports describing the detailed expression patterns, or the functional studies, of many genes during cranial development. Many examples of gene mutations that affect cranial morphology have been accumulating [7]; however, the developmental process of the cranium is so complicated that many questions remain unanswered.

## Conclusion

Geometric morphometrics revealed that the cranial characteristics of each species and subspecies were clearly distinguishable. *M. spretus*, a different species from the other 6 strains, has an extremely slender cranium compared with the other strains. Hallgrímsson et al argued that A/WySnJ mice exhibit altered facial morphology which results from a reduction in the growth of the maxillary process during formation of the face, and that this is relevant to evolutionary changes in facial prognathism in nonhuman primates and in human evolution [21]. *M. spretus *has a long maxillary and is the opposite to A/WySnJ mice. *M. spretus *diverged from *M. musculus *about 3 million years ago and developed into a different mouse species. Our data also suggests that genetically close strains do not always possess morphologically similar crania; the mean cranial shape of C3H and CBA were more similar to MSM/Ms, which is derived from *M. m. molossinus *[20], than to either C57BL/6J, BALB, or ICR which are paradoxically genetically closer.

## Methods

### Specimens

The mice used in this study were all 12 weeks old. C57BL/6J, BALB/cA, C3H/HeJ, and ICR mice were purchased from Clea Japan Inc. (Meguro Ward, Tokyo, Japan). CBA/JNCr mice were purchased from Charles River Laboratories Japan Inc. (Kohoku Ward, Yokohama, Japan). MSM/Ms mice and *Mus spretus *were obtained from the Riken BioResource Center (Tsukuba, Ibaraki, Japan). After euthanasia, the mice were skinned and the crania were removed and cleaned. All animal experiments were carried out with the approval of the Ethical Committee at the Center for Animal Resources and Development, Kumamoto University (D-18-090, A-19-154).

### Linear Distance Measurement

We measured distances between 11 landmarks directly using digital calipers (Fig. 2). Statistical analyses were performed using the ystat 2000 Program.

### Morphometrics

Crania were photographed in dorsal view using a Keyence Digital Microscope and 14 landmarks were digitized (Fig. 3). Relative warp analysis was performed using the tpsRelw Program.

### Regression Analysis Between Size and Shape

The calculation of centroid size (CS) was done in the CoordGen6f Program, part of the Integrated Morphometrics Programs (IMP), produced in Matlab6 [29]. Univariate regression analysis was done in Microsoft Excel.

### Cluster Analysis

Cluster analyses (unweighted pair-group average [UPGA]) were performed for interspecies distance between their mean shapes (acquired with the tpsRelw Program) on a scatter plot of relative warp (RW) 1 versus RW2, and a dendrogram was constructed.

## Authors' contributions

MK is responsible for executing all work involved in this paper. KY conceived of this study and supervised the data analyses. Both authors read and approved the final manuscript.
